# Supplementary material for: Self-Adaptive Flask-like Nanomotors Based on Fe3O4 Nanoparticles to a Physiological pH
Source: Nanomaterials (Basel). 2022 Jun 15;12(12):2049. doi: 10.3390/nano12122049 (PMC9229371; doi:10.3390/nano12122049)
Supplement: Supplementary file 1 [file nanomaterials-12-02049-s001.zip › nanomaterials-1731164-supplementary.pdf]

nanomaterials

MDPI

Supplementary Materials

Self-Adaptive Flask-Like Nanomotors Based on Fe_3O_4 Nanoparticles to a Physiological PH

Tianyu Gao [1], Jinwei Lin [2], Leilei Xu [1,*] and Jianguo Guan [1,2,*]

[1] State Key Laboratory of Advanced Technology for Materials Synthesis and Processing, School of Materials Science and Engineering, Wuhan University of Technology, Wuhan 430070, China; gty@whut.edu.cn.
[2] State Key Laboratory of Advanced Technology for Materials Synthesis and Processing, International School of Materials Science and Engineering, Wuhan University of Technology, Wuhan 430070, China; linjinwei@whut.edu.cn.
* Correspondence: xull@whut.edu.cn (L.X.); guanjg@whut.edu.cn (J.G.)

Supplementary Table:

Table S1. Typical parameters used in the simulations.

Parameters	Description	Values	Units
S_Fe3O4 *	surface molar concentration	7.14×10^{-5}	mol/m^2
Zeta_flask	Zeta potential of FCNMs	-0.057	V
D_H	diffusivity of H^+	9.31×10^{-9}	m^2/s
D_OH	diffusivity of OH^-	5.3×10^{-9}	m^2/s
D_• OH	diffusivity of • OH	2.3×10^{-9}	m^2/s
D_O2	diffusivity of O_2	1.97×10^{-9}	m^2/s
D_$C_6H_8O_7$	diffusivity of $C_6H_8O_7$	0.66×10^{-9}	m^2/s
D_$C_6H_7O_7$	diffusivity of $C_6H_7O_7^-$	0.98×10^{-9}	m^2/s

* Calculation of surface molar concentration of Fe_3O_4 NPs (S_{Fe3O4})

From the TEM images (Figure 2b), it can be estimated that the average volume of individual flask-like carbonaceous carrier (FCC, only the carbon shell is counted, and the cavity is not counted) is $V_{FCC}= 2.76\times10^{-20}$ m^3 and the internal surface area of individual FCC (only the bottom is counted, and the neck is not counted) is $S_{FCC} = 3.35\times10^{-13}$ m^2.

Based on the density of amorphous carbon ($\rho_C=1.8\times10^6$ g m^{-3}), the weight of individual FCC (m_{FCC}) is calculated as follows:

$$m_{FCC} = V_{FCC} \times \rho_C = 4.97 \times 10^{-14} g \quad (1)$$

Based on the results of TG analysis (Figure 2e) and the relative molecular weight of Fe_3O_4 NP (Mr_{Fe3O4}=231.54 g/mol), the number of FCCs (N_{FCCs}) and the molar amount of Fe_3O_4 NPs contained in each FCCs (n_{Fe3O4}) can be calculated as follows:

$$N_{FCCs} = \frac{m'_{FCCs}}{m_{FCC}} = 7.08 \times 10^{10} \quad (2)$$

$$n_{Fe3O4} = \frac{m_{Fe3O4}}{N_{FCCs} \times Mr_{Fe3O4}} = 2.39 \times 10^{-17}\, mol \quad (3)$$

Assuming that the Fe_3O_4 NPs is evenly distributed at the bottom of the bottle, the surface molar concentration of the Fe_3O_4 NPs (S_{Fe3O4}) can be calculated as follows:

$$S_{Fe3O4} = \frac{n_{Fe3O4}}{S_{FCC}} = 7.14 \times\ 10^{-5}\, mol/m^2 \quad (4)$$

Table S2. Average size, saturation magnetization (Ms) and relative peroxidase-like activity of as-prepared different Fe_3O_4@OA NPs.

Samples	Average size (nm)	Ms (emu/g)	Relative activity (%)
MNP-1	4.62	33.9	97.5
MNP-2(5)	6.85	50.5	69.8
MNP-3	7.37	59.1	60.0
MNP-4	4.19	40.9	100
MNP-6	7.73	61.7	59.9
MNP-7	8.66	65.3	40.9
MNP-8	2.94	7.02	28.4
MNP-9	9.52	71.3	64.1
MNP-10	6.91	54.5	68.5
MNP-11	6.06	49.9	83.8

Supplementary Figure:

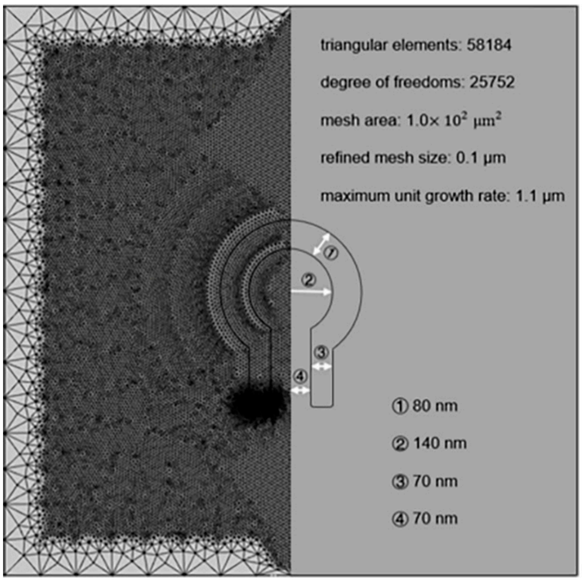

Figure S1. Meshing conditions of the theoretical model.

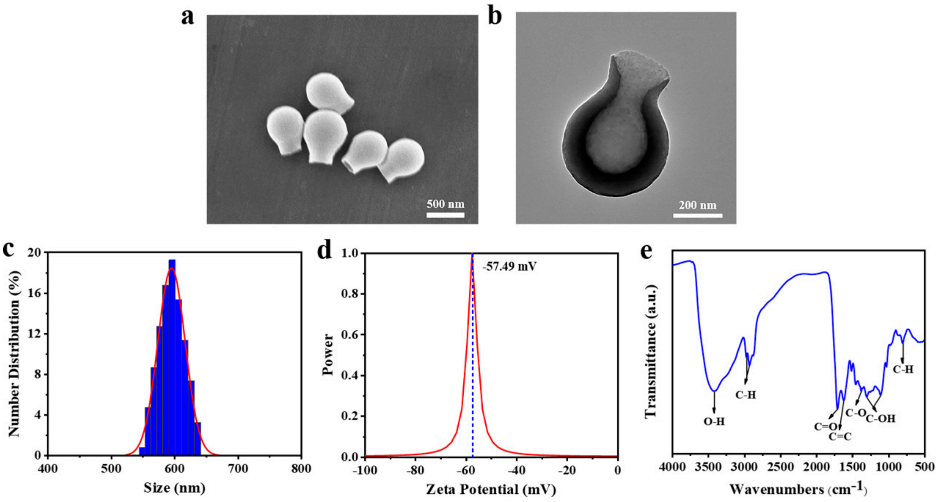

Figure S2. Characterizations of the FCCs. (**a**) SEM image of the FCCs. (**b**) TEM image of a typical FCC. (**c**) Zeta potential, (**d**) hydrodynamic size analysis and (e) FTIR spectra of the FCCs.

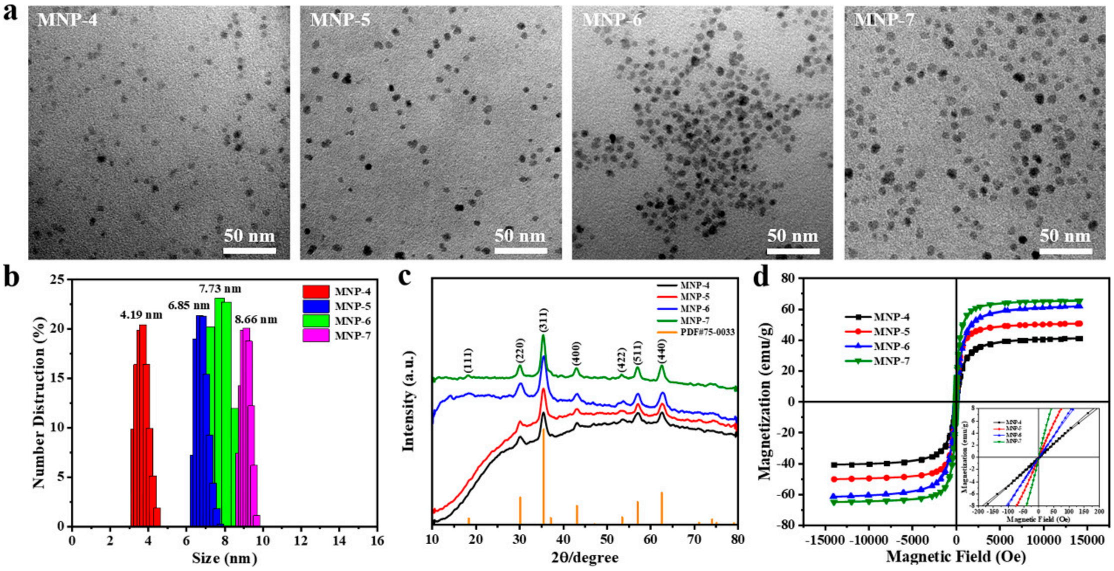

Figure S3. Characterizations of the Fe_3O_4@OA NPs synthesized at different aging temperatures (MNP-1, 200 °C; MNP-2, 220 °C; MNP-3, 240 °C). (**a**) TEM images, (**b**) hydrodynamic size analysis, (**c**) XRD patterns, and (**d**) hysteresis loops of the Fe_3O_4@OA NPs.

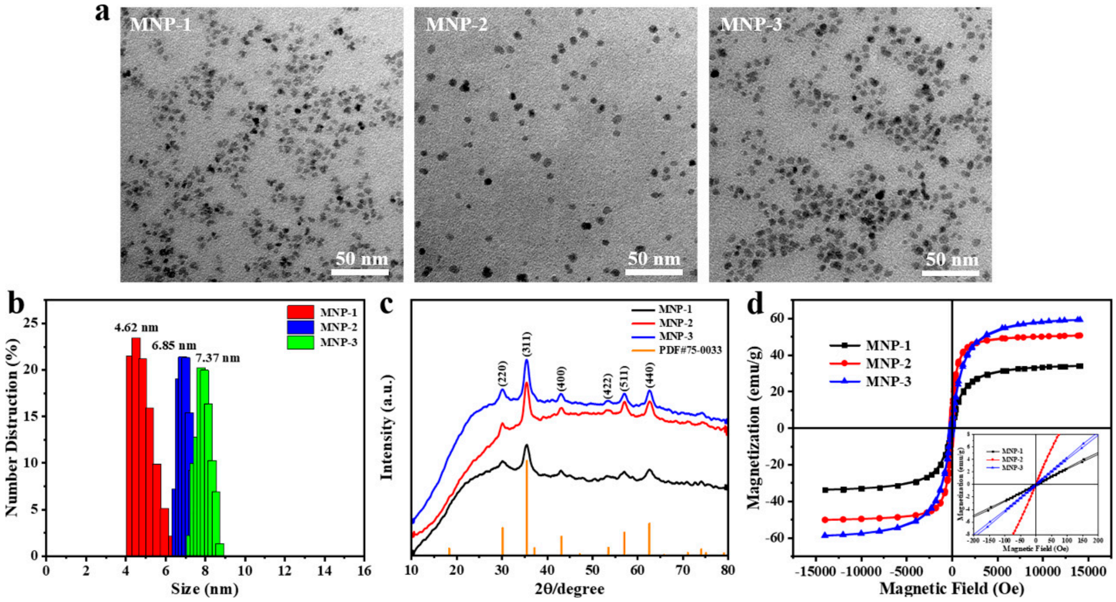

Figure S4. Characterizations of the Fe_3O_4@OA NPs synthesized at different aging time (MNP-4, 10 min; MNP-5, 30 min; MNP-6, 60 min; MNP-7, 90 min). (**a**) TEM images, (**b**) hydrodynamic size analysis, (**c**) XRD patterns, and (**d**) hysteresis loops of the Fe_3O_4@OA NPs.

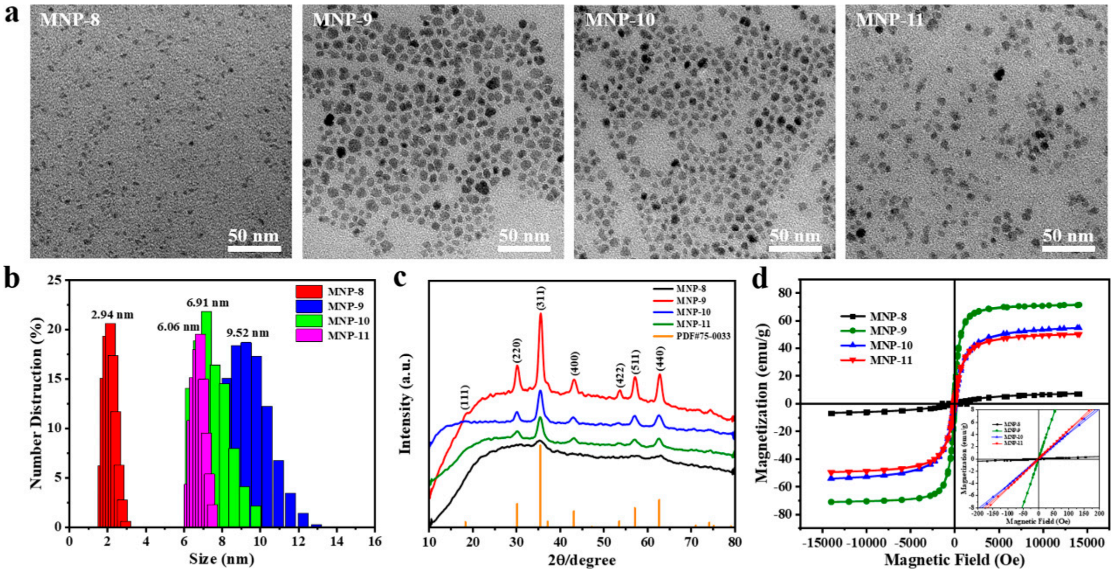

Figure S5. Characterizations of the Fe_3O_4@OA NPs synthesized at different raw material molar ratios between oleyl alcohol and oleic acid (MNP-8, 12 : 12; MNP-9, 16 : 8; MNP-10, 20 : 4; MNP-11, 24 : 0). (**a**) TEM images, (**b**) hydrodynamic size analysis, (**c**) XRD patterns, and (**d**) hysteresis loops of the Fe_3O_4@OA NPs.

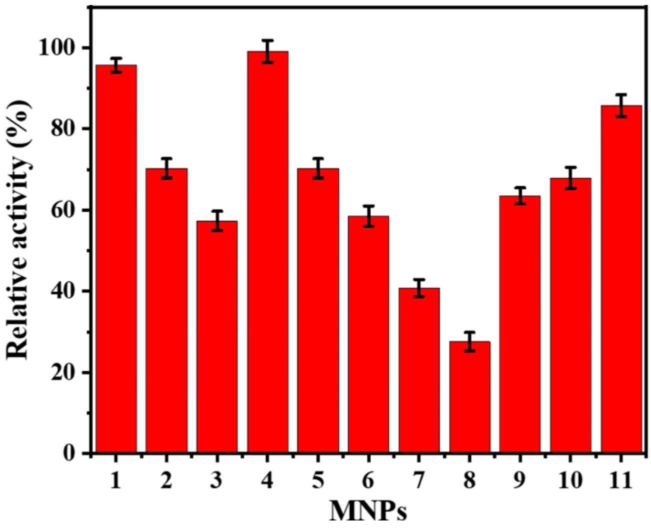

Figure S6. Relative peroxidase-like activities of the as-synthesized Fe_3O_4@OA NPs calculated by taking the group with the highest activity as 100%.

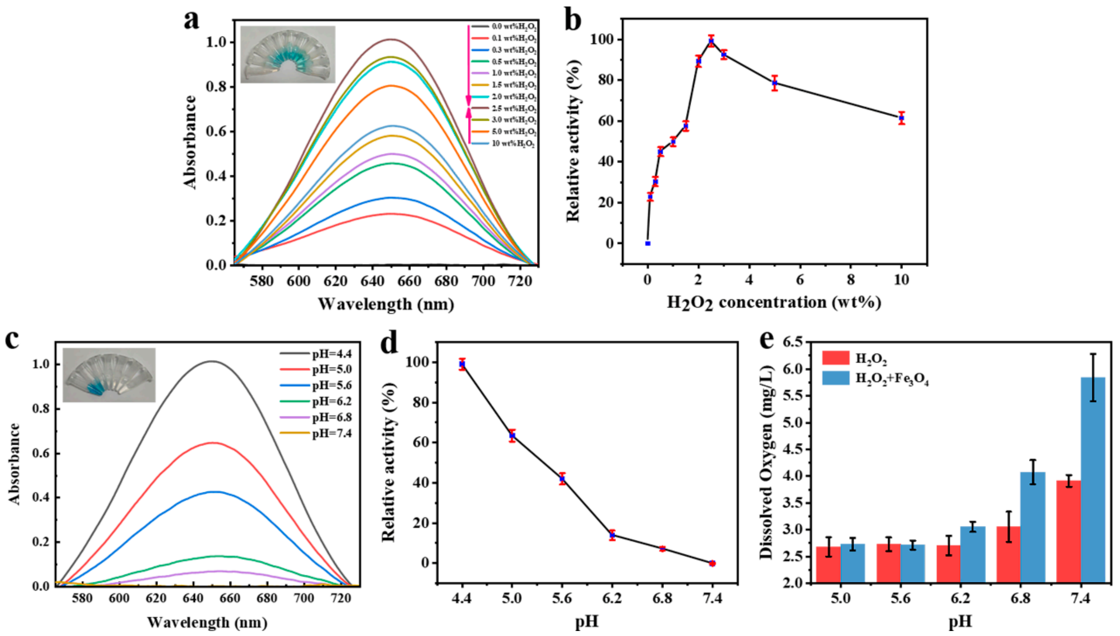

Figure S7. Characterizations of oil-soluble Fe_3O_4@OA and water-soluble Fe_3O_4@DMSA NPs. (**a**) TEM image of Fe_3O_4@DMSA NPs. (**b**) Fe 2p XPS spectra of the Fe_3O_4@DMSA NPs. (**c**) DLS, (**d**) VSM, and (**e**) FITR of Fe_3O_4@OA and Fe_3O_4@DMSA NPs. (**f**) Zeta potentials of the Fe_3O_4@DMSA NPs at different pH values.

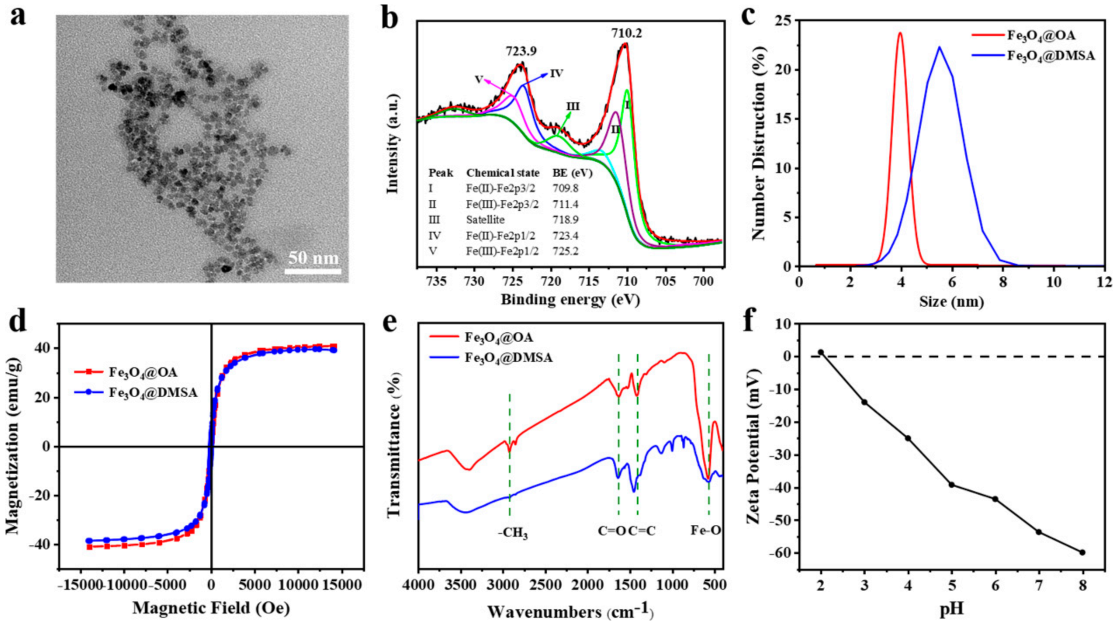

Figure S8. Verifications of dual enzyme-like activities of the Fe_3O_4@DMSA NPs. (**a**) UV-Vis spectra of the TMB-H_2O_2 system at different H_2O_2 concentrations. (**b**) Relative peroxidase-like activity of the Fe_3O_4@DMSA NPs at different H_2O_2 concentrations calculated by taking the activity at 2.5 wt% as 100%. (**c**) UV-Vis spectra of TMB-H_2O_2 system at different pH values. (**d**) Relative catalase-like activity of the Fe_3O_4@DMSA NPs at different pH values calculated by taking the activity at pH 7.4 as 100%. (**e**) Dissolved oxygen analysis of the as-prepared FCNMs moving in 2.5 wt% H_2O_2 aqueous solution at different pH values.

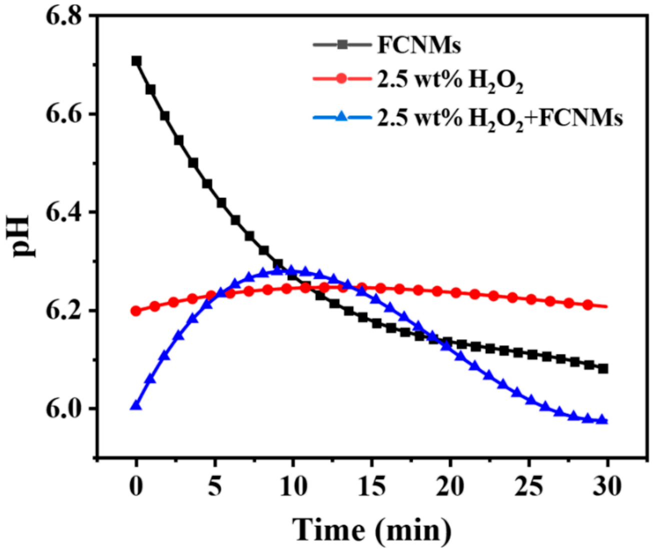

Figure S9. Verification of OH^- product by monitoring the change of pH values of pure 2.5 wt% H_2O_2 aqueous solution, FCNMs aqueous solution and their mixed solutions in 30 min.

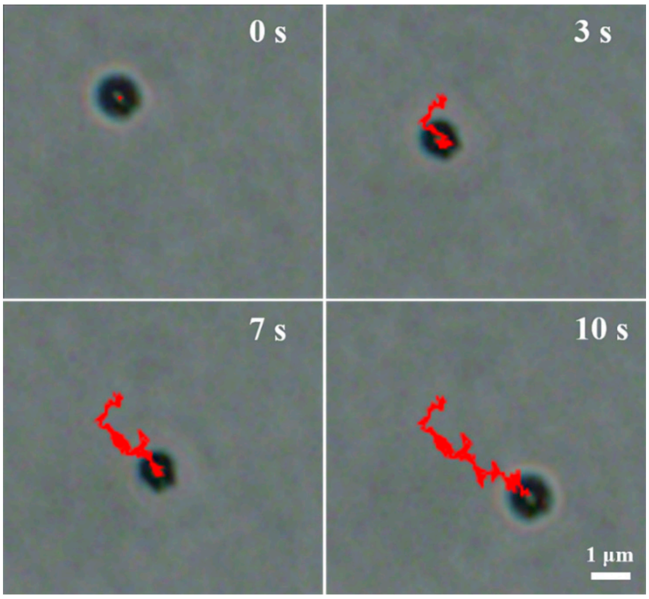

Figure S10. Time-lapse images monitored the motion direction of a comparable FCNM with a larger size of ~1 µm in 2.5 wt% H_2O_2 aqueous solution at pH 4.4.

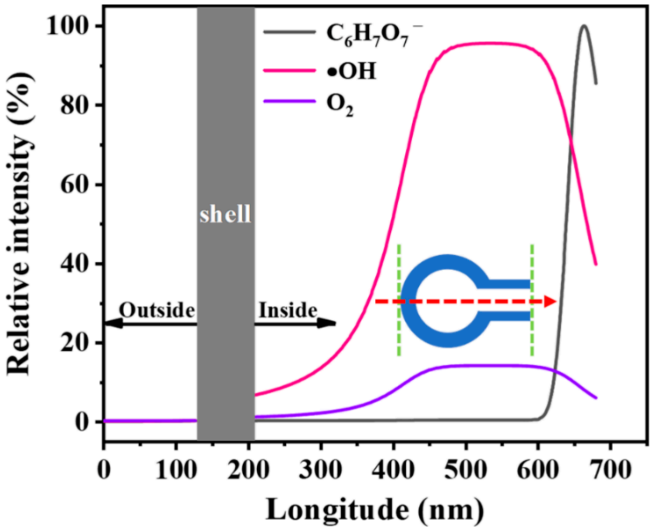

Figure S11. Concentration gradient distributions of •OH, $C_6H_7O_7^-$ and O_2 on the symmetric axis of the FCNMs.

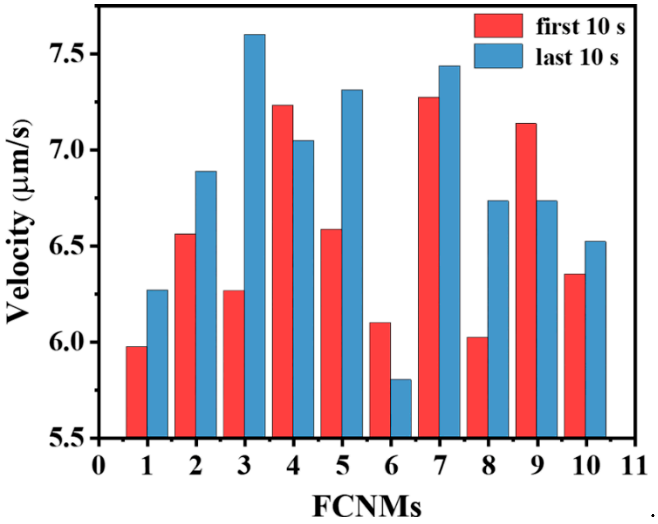

Figure S12. A discrete distribution of the average velocities of different chemotactic FCNMs in the first 10 s and last 10 s. .

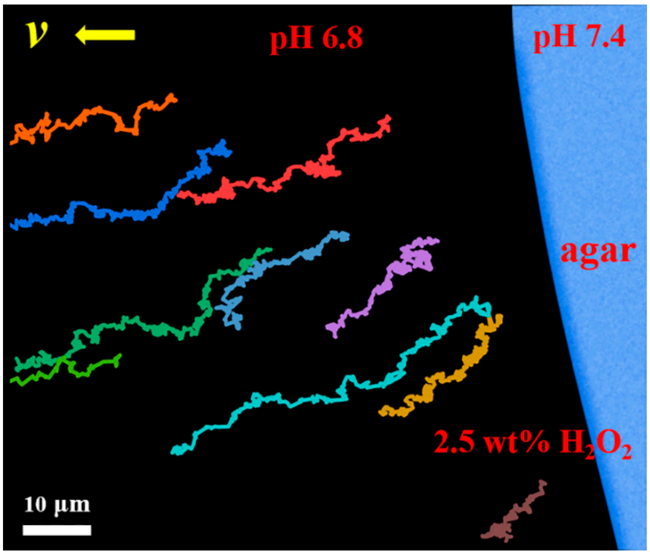

Figure S13. Chemotactic track trajectories of the FCNMs in 2.5 wt% H_2O_2 aqueous solution with a right-to-left gradually decreasing pH gradient in 30 s.

Supplementary Video:

Video S1. The FCNM moving in 0 wt% H_2O_2 aqueous solution.

Video S2. The FCNM moving in 2.5 wt% H_2O_2 aqueous solution at pH 5.6.

Video S3. The FCNM moving in 2.5 wt% H_2O_2 aqueous solution at pH 6.8.

Video S4. The FCNM with a larger size moving in 2.5 wt% H_2O_2 aqueous solution at pH 4.4 for observing its motion direction.

Video S5. The FCNM moving in 2.5 wt% H_2O_2 aqueous solution.

Video S6. The FCNMs moving in 2.5 wt% H_2O_2 aqueous solution with a left-to-right gradually decreasing pH gradient.

Video S7. The FCNMs moving in 2.5 wt% H_2O_2 aqueous solution with a right-to-left gradually decreasing pH gradient.
